# Supplementary material for: Innovative Mitral Valve Repair Using a Novel Automated Suturing System: Preliminary Data
Source: Medicina (Kaunas). 2024 Jul 15;60(7):1138. doi: 10.3390/medicina60071138 (PMC11278705; doi:10.3390/medicina60071138)
Supplement: Supplementary file 1 [file medicina-60-01138-s001.zip › Supplementary Data_Worksheet.pdf]

Modified ROMAN ARCH – MiStitch

SPECIMEN NUMBER ______________

SURGEON ______________

Commissural width ______________ [mm]

P2 segment width ______________ [mm]

Number of cut chordal tendons ______________ [n]

Width of the flail leaflet ______________ [mm]

Anticipated technique: “wrapping” of PMVL (atraumatic bites only) O yes O no

Total time (from first bite till suture fixation with MiKnot): _______________[mm:ss]

Time for suture placement (first bite, till suture cut): _______________[mm:ss]

Time for fixation (suture cut, till MiKnot fixation): _______________[mm:ss]

Number of leaflets bites ______________ [n]

Comment: ____

Suture deployment as desired (depth and location) O yes O no

Entanglement or other problems with the placement of the suture O yes O no

Knot Fixation as desired O yes O no

Comment: ____

Total length of ePTFE suture (after cutting) ______________ [mm]

Describe the overall feeling from placement: [tick the right box]
(0% unsatisfactory – 25% rather unsatisfactory – 50% neutral – 75% rather satisfactory – 100% very satisfactory):

[0%] - - - - - - - - - - **[25%]** - - - - - - - - - - **[50%]** - - - - - - - - - - **[75%]** - - - - - - - - - - **[100%]**
